# Supplementary material for: Guaianolide Sesquiterpene Lactones from Centaurothamnus maximus
Source: Molecules. 2021 Apr 3;26(7):2055. doi: 10.3390/molecules26072055 (PMC8038342; doi:10.3390/molecules26072055)
Supplement: Supplementary file 1 [file molecules-26-02055-s001.pdf]

Supplementary Materials

Guaianolide Sesquiterpene Lactones from *Centaurothamnus maximus*

Taha A. Hussien [1,†], Tarik A. Mohamed [2,†], Abdelsamed I. Elshamy [3], Mahmoud F. Moustafa [4,5], Hesham R. El-Seedi [6,7,*], Paul W. Pare [8] and Mohamed-Elamir F. Hegazy [2,*]

[1] Pharmacognosy Department, Faculty of Pharmacy, Sphinx University, New Assiut City, Assiut 10, Egypt; thussien71@yahoo.com

[2] Chemistry of Medicinal Plants Department, National Research Centre, 33 El-Bohouth St., Dokki, Giza 12622, Egypt; ta.mourad@nrc.sci.eg

[3] Department of Natural Compounds Chemistry, National Research Centre, 33 El-Bohouth St., Dokki, Giza 12622, Egypt; ai.el-shamy@nrc.sci.eg

[4] Department of Biology, College of Science, King Khalid University, Abha 61421, Saudi Arabia; mfmostfa@kku.edu.sa

[5] Department of Botany & Microbiology, Faculty of Science, South Valley University, Qena 83523, Egypt

[6] Department of Molecular Biosciences, The Wenner-Gren Institute, Stockholm University, S-106 91 Stockholm, Sweden

[7] International Research Center for Food Nutrition and Safety, Jiangsu University, Zhenjiang 212013, China

[8] Department of Chemistry, Faculty of Science, Menoufia University, 32512 Shebin El-Kom, Egypt

[9] Department of Chemistry & Biochemistry, Texas Tech University, Lubbock, TX 79409, USA; paul.pare@ttu.edu

[10] Department of Pharmaceutical Biology, Institute of Pharmaceutical and Biomedical Sciences, Johannes Gutenberg University, Staudinger Weg 5, 55128 Mainz

* Correspondence: hesham.elseedi@su.se (H.R.E.-S.); me.fathy@nrc.sci.eg (M.-E.F.H.); Tel.: +46-73-566-8234 (H.R.E.-S.); Tel.: +2-033-371-635 (M.-E.F.H.)

† These authors contributed equally to this work.

Supporting data **Page**

S1:^{1}H NMR ($CDCl_3$, 500 MHz) of **1** …………………………….. 3

S2:^{13}C NMR ($CDCl_3$, 125 MHz) of **1** …………………………… 4

S3:DEPT of **1** …………………………………………………... 5

S4:HSQC of **1** ………………………………………………….. 6

S5:HMBC of **1**………………………………………………… 7

S6:^{1}H ^{1}H COSY of **1**………………………………………… 8

S7:NOESY**1**……………………………………………………... 9

S8: HRCIMS**1**…………………………………………………... 10

S9:^{1}H NMR ($CDCl_3$, 500 MHz) of **2** …………………………….. 11

S10:^{13}C NMR ($CDCl_3$, 125 MHz) of **2** …………………………… 12

S11:DEPT of **2** …………………………………………………... 13

S12:HSQC of **2** ………………………………………………….. 14

S13:HMBC of **2**………………………………………………… 15

S14:^{1}H ^{1}H COSY of **2**………………………………………… 16

S15:NOESY **2**……………………………………………………... 17

S16: HRCIMS**2**…………………………………………………... 18

S17:^{1}H NMR ($CDCl_3$, 500 MHz) of **3** …………………………….. 19

S18:^{13}C NMR ($CDCl_3$, 125 MHz) of **3** …………………………… 20

S19:^{1}H NMR ($CDCl_3$, 500 MHz) of **4**…………………………….. 21

S20:^{13}C NMR ($CDCl_3$, 125 MHz) of **4** …………………………… 22

S21:^{1}H NMR ($CDCl_3$, 500 MHz) of **5** …………………………….. 23

S22:^{13}C NMR ($CDCl_3$, 125 MHz) of **5** …………………………… 24

S23:^{1}H NMR ($CDCl_3$, 500 MHz) of **6** …………………………….. 25

S24:^{13}C NMR ($CDCl_3$, 125 MHz) of **6** …………………………… 26

S25:^{1}H NMR ($CDCl_3$, 500 MHz) of **7** …………………………….. 27

S26:^{13}C NMR ($CDCl_3$, 125 MHz) of **7** …………………………… 28

S27:^{1}H NMR ($CDCl_3$, 500 MHz) of **8** …………………………….. 29

S28:^{1}H NMR ($CDCl_3$, 500 MHz) of **9** …………………………….. 31

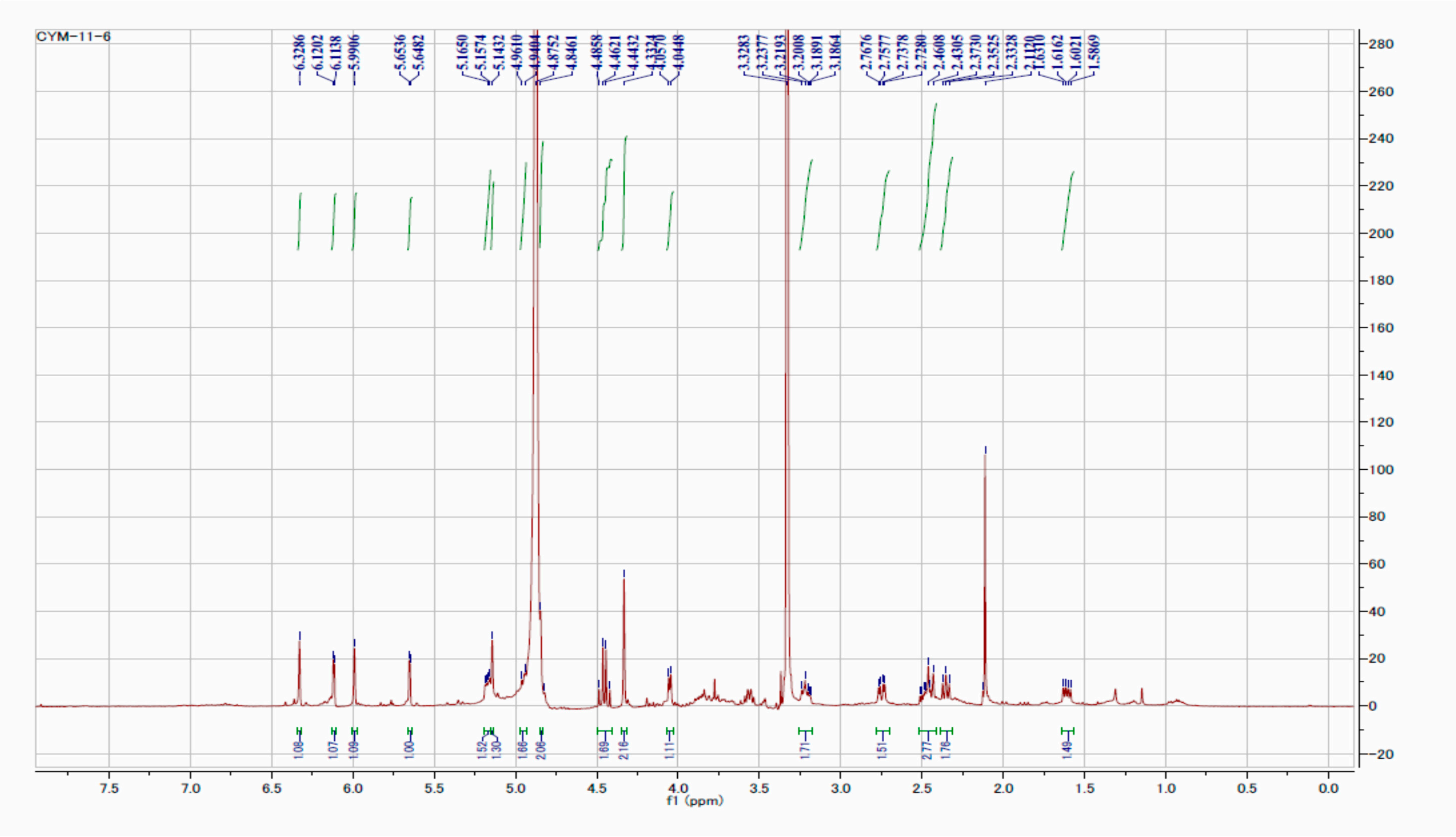

S1: ¹H NMR ($CDCl_3$, 500 MHz) of **1**

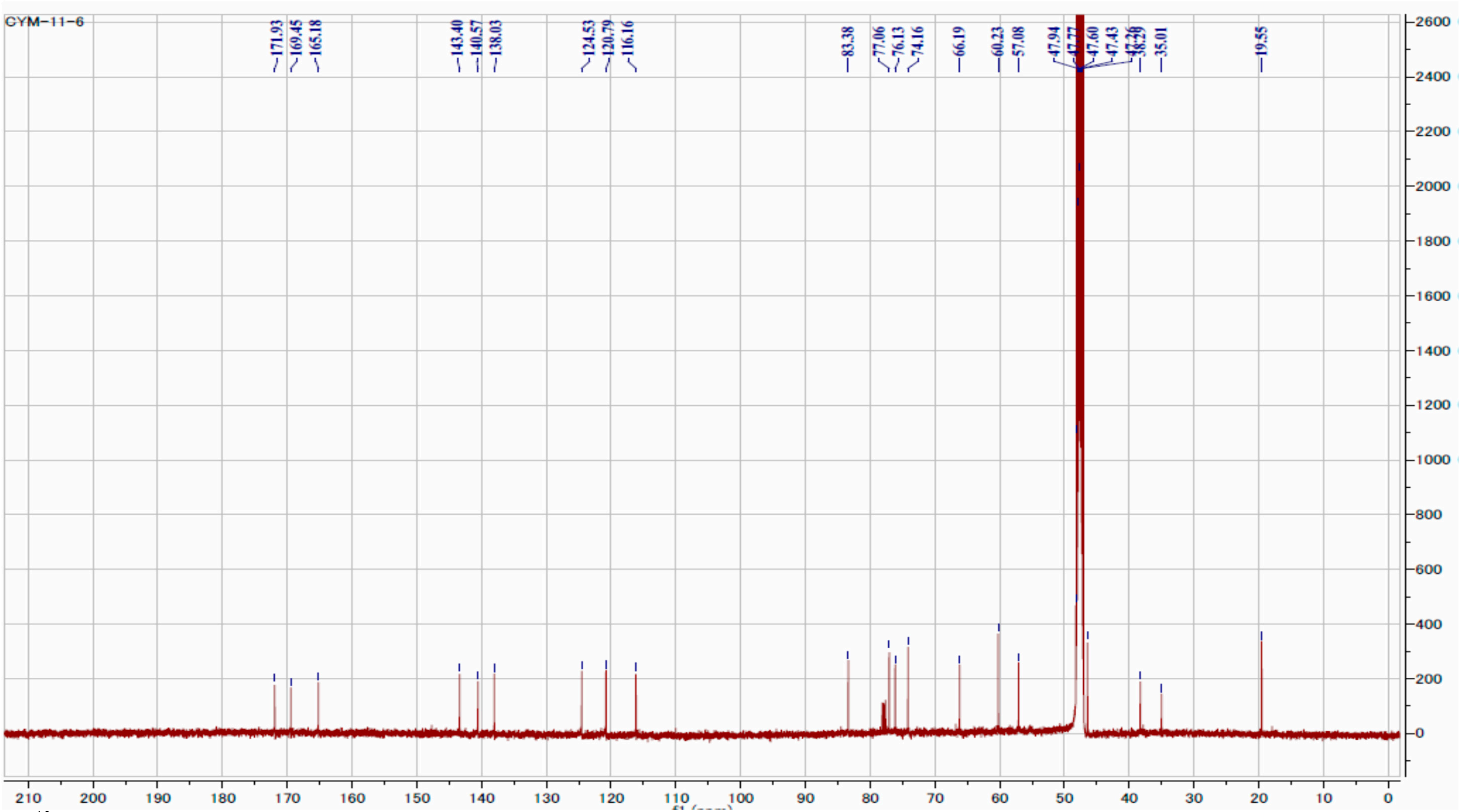

S2:^{13}C NMR (CDCl3, 125 MHz) of **1**

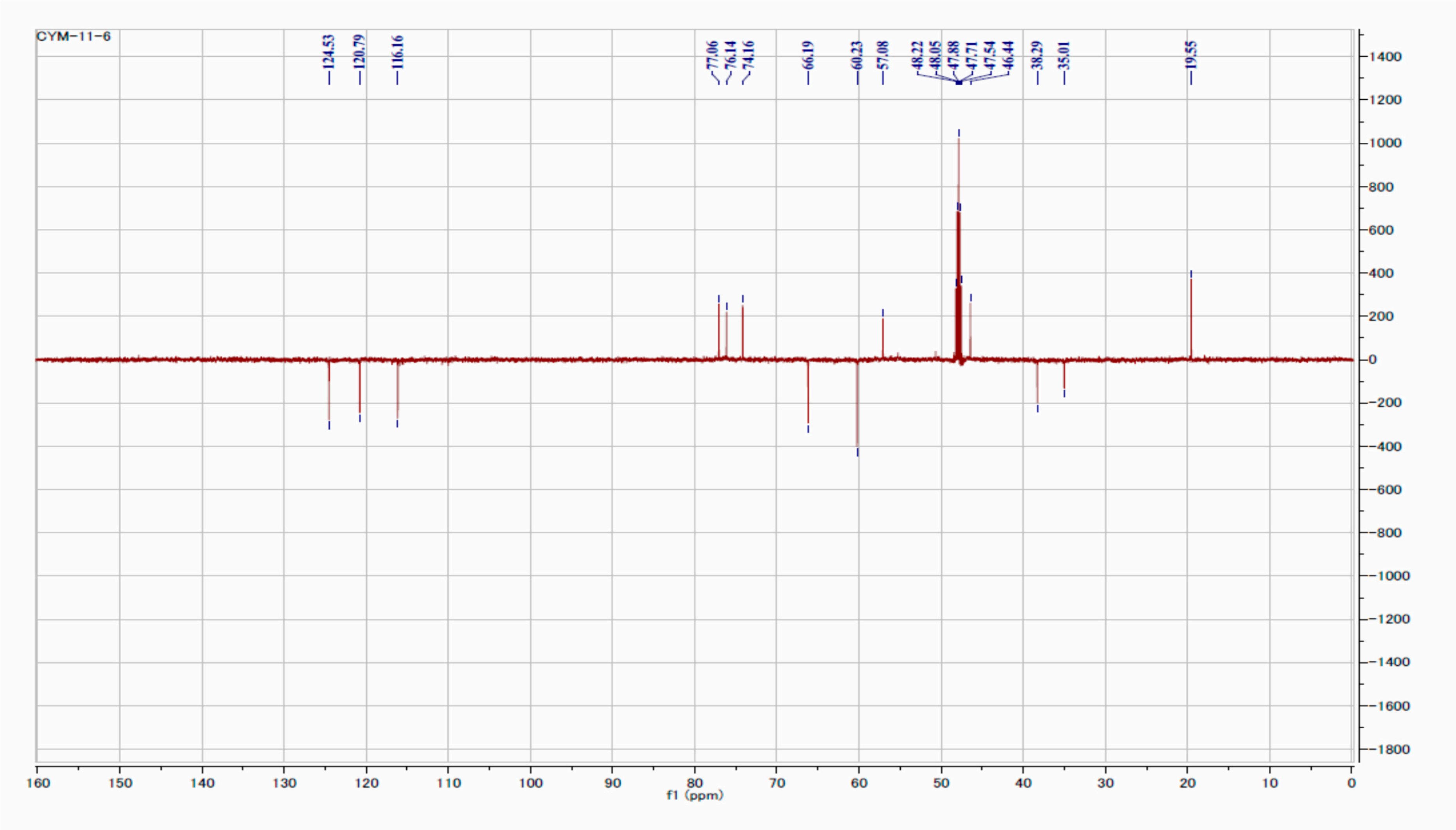
CYM-11-6
124.53
120.79
116.16
77.06
76.14
74.16
66.19
60.23
57.08
48.22
48.05
47.88
47.71
47.54
46.44
38.29
35.01
19.55
f1 (ppm)

S3:DEPT of **1**

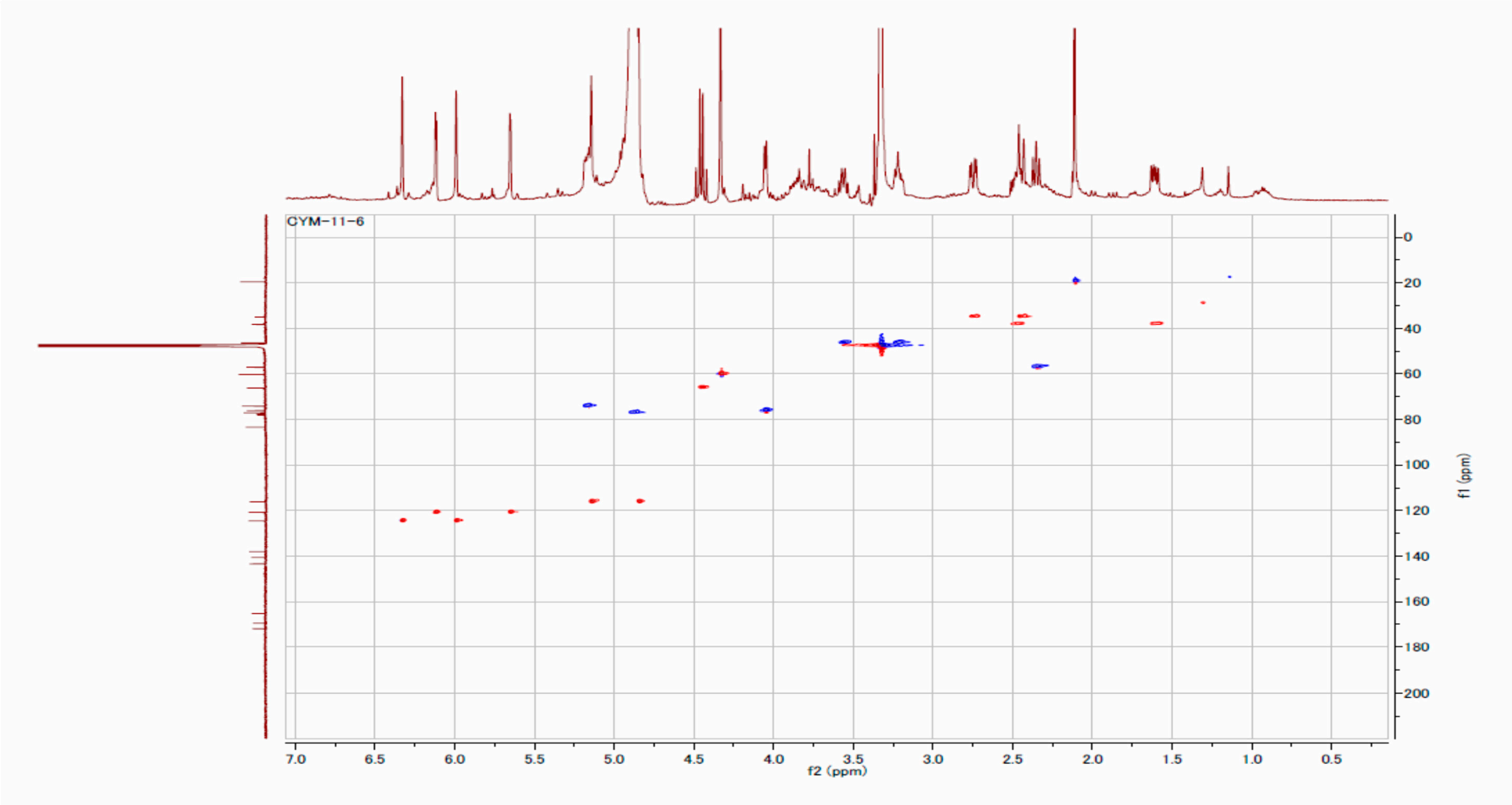
CYM-11-6
7.0
6.5
6.0
5.5
5.0
4.5
4.0
3.5
3.0
2.5
2.0
1.5
1.0
0.5
f2 (ppm)
0
20
40
60
80
100
120
140
160
180
200
f1 (ppm)

S4:HSQC of **1**

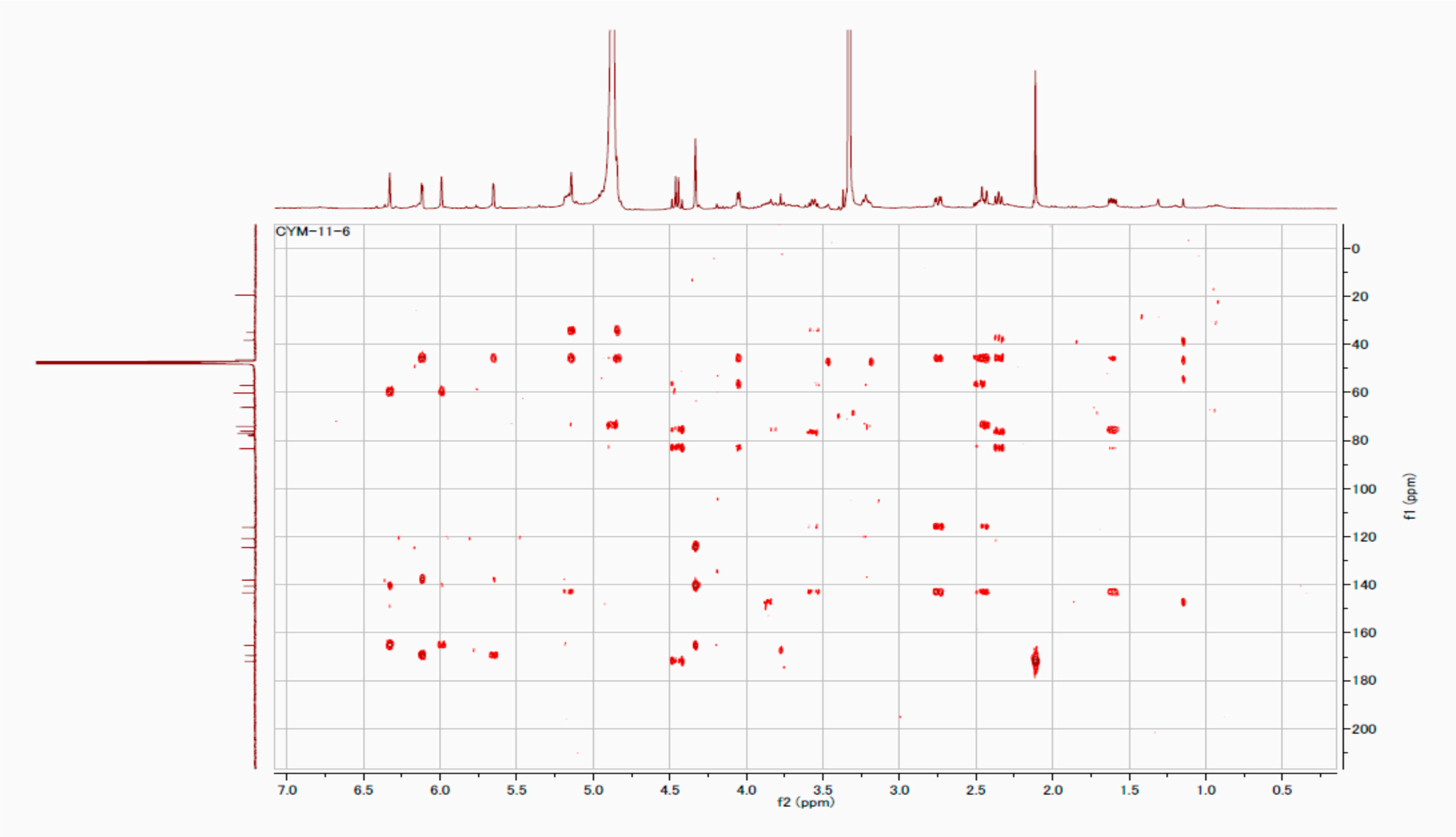

S5:HMBC of **1**

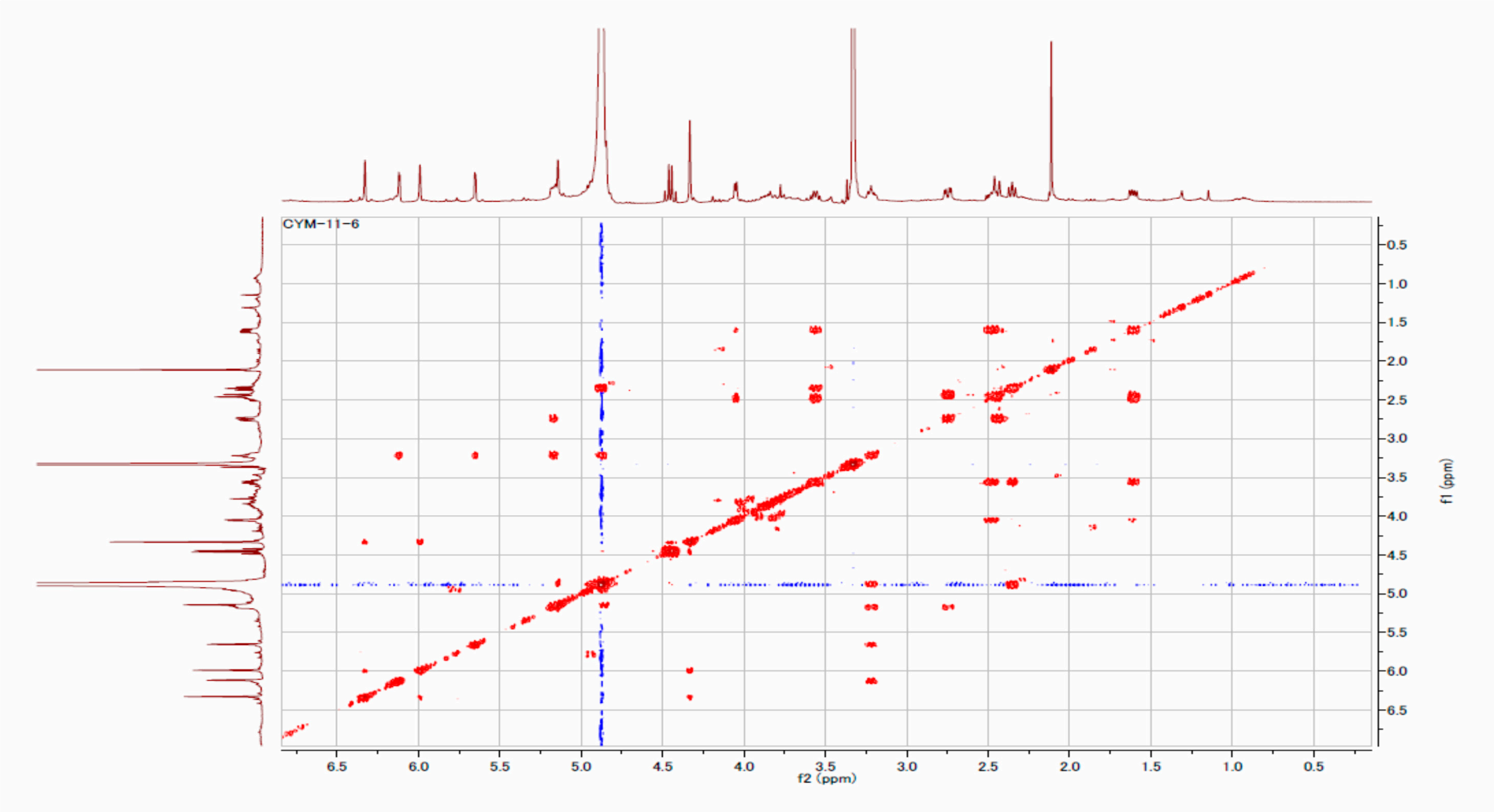

S6:^{1}H ^{1}H COSY of **1**

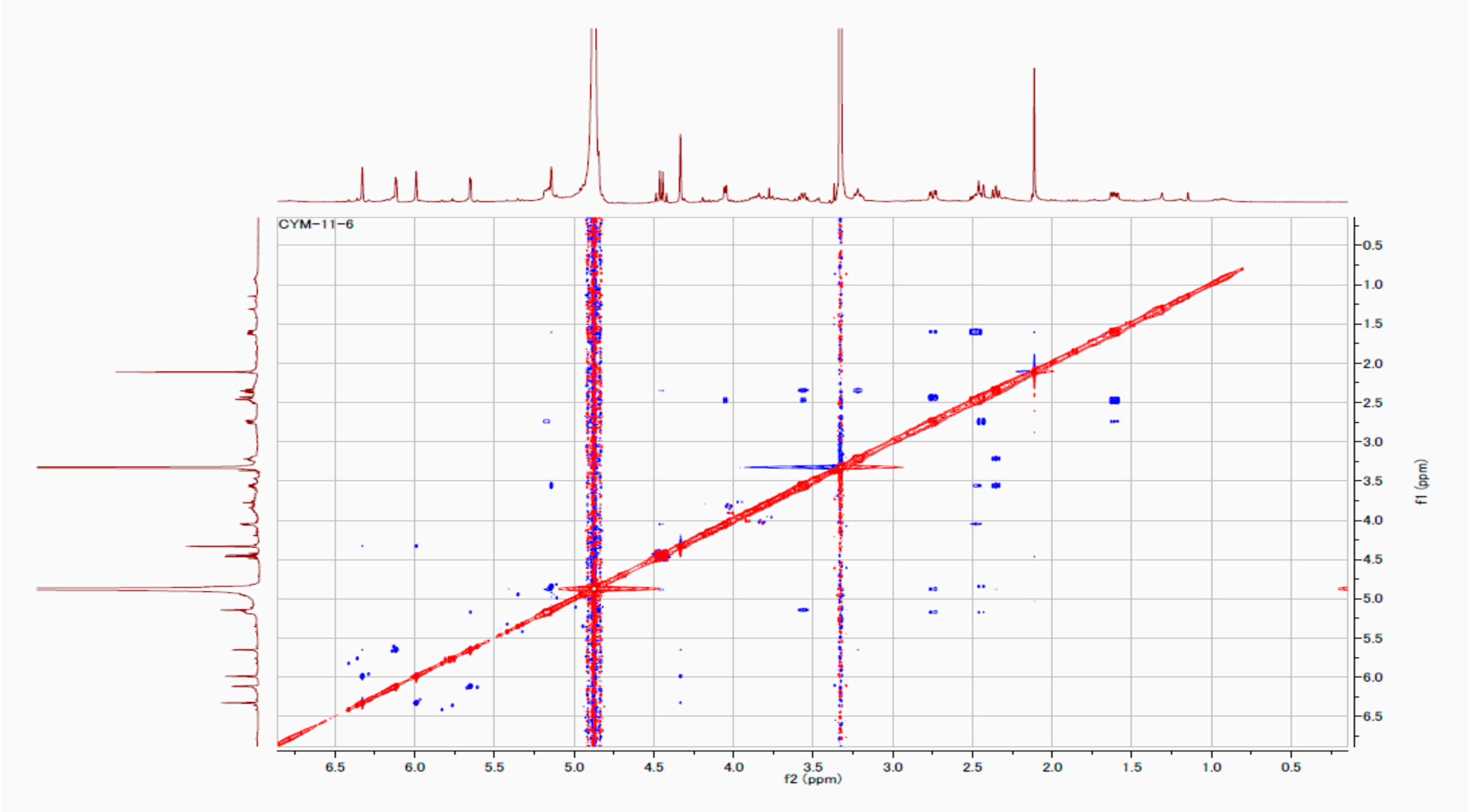

S7:NOESY of **1**

Sample : CyM-11-6
Note : MStation
Inlet : Direct Ion Mode : CI+
RT : 2.11 min Scan# : 56
Elements : C 150/0, H 250/0, O 50/0
Mass Tolerance : 5mmu
Unsaturation (U.S.) : 0.0 - 15.0

	Observed m/z	Int%	Err[ppm / mmu]	U.S.	Composition
1	423.1655	18.79	-0.0 / -0.0	8.5	C21 H27 O9

S8: HRCI/mas of **1**

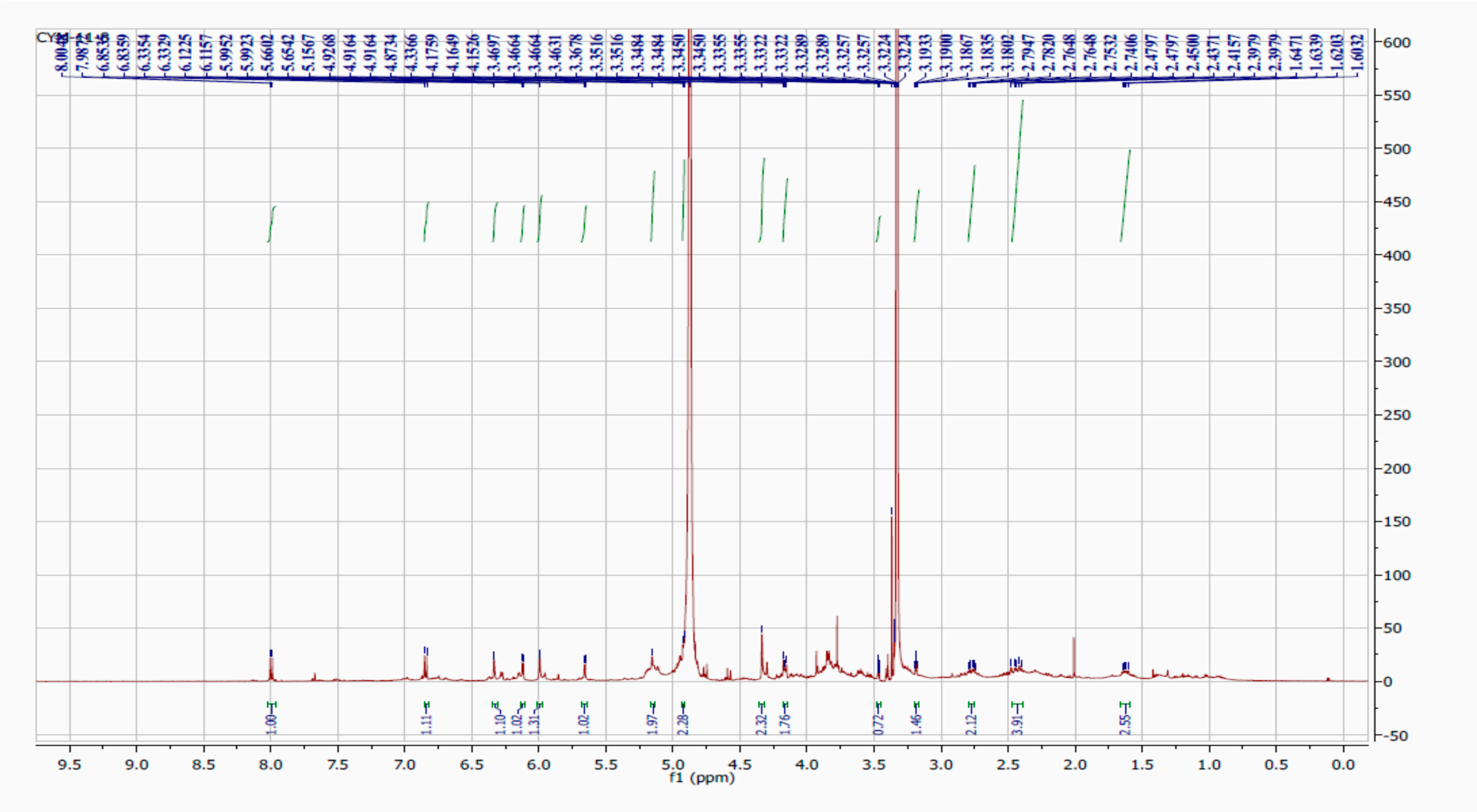

S9:^{1}H NMR ($CDCl_3$, 500 MHz) of **2**

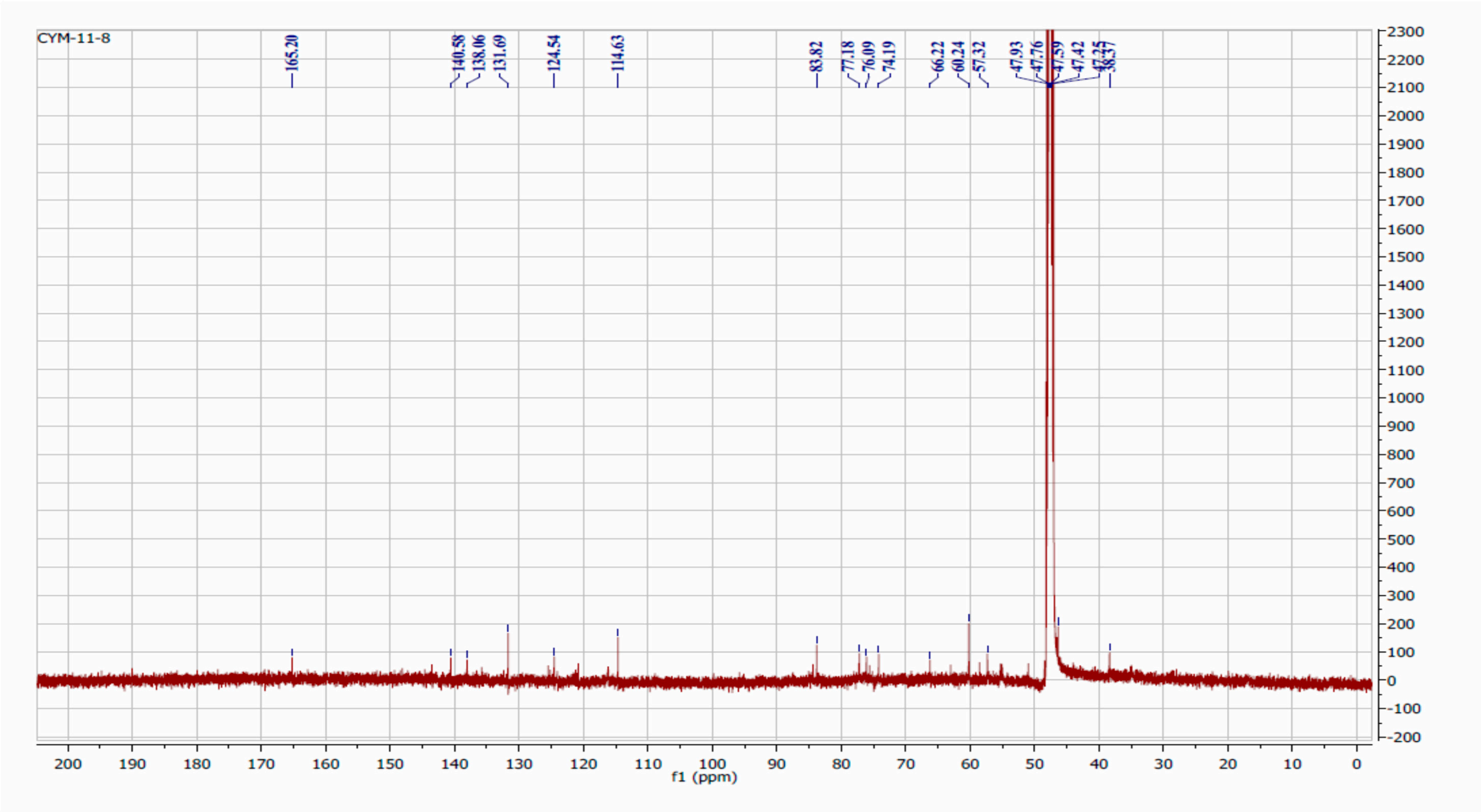

S10:^{13}C NMR (CDCl3, 125 MHz) of **2**

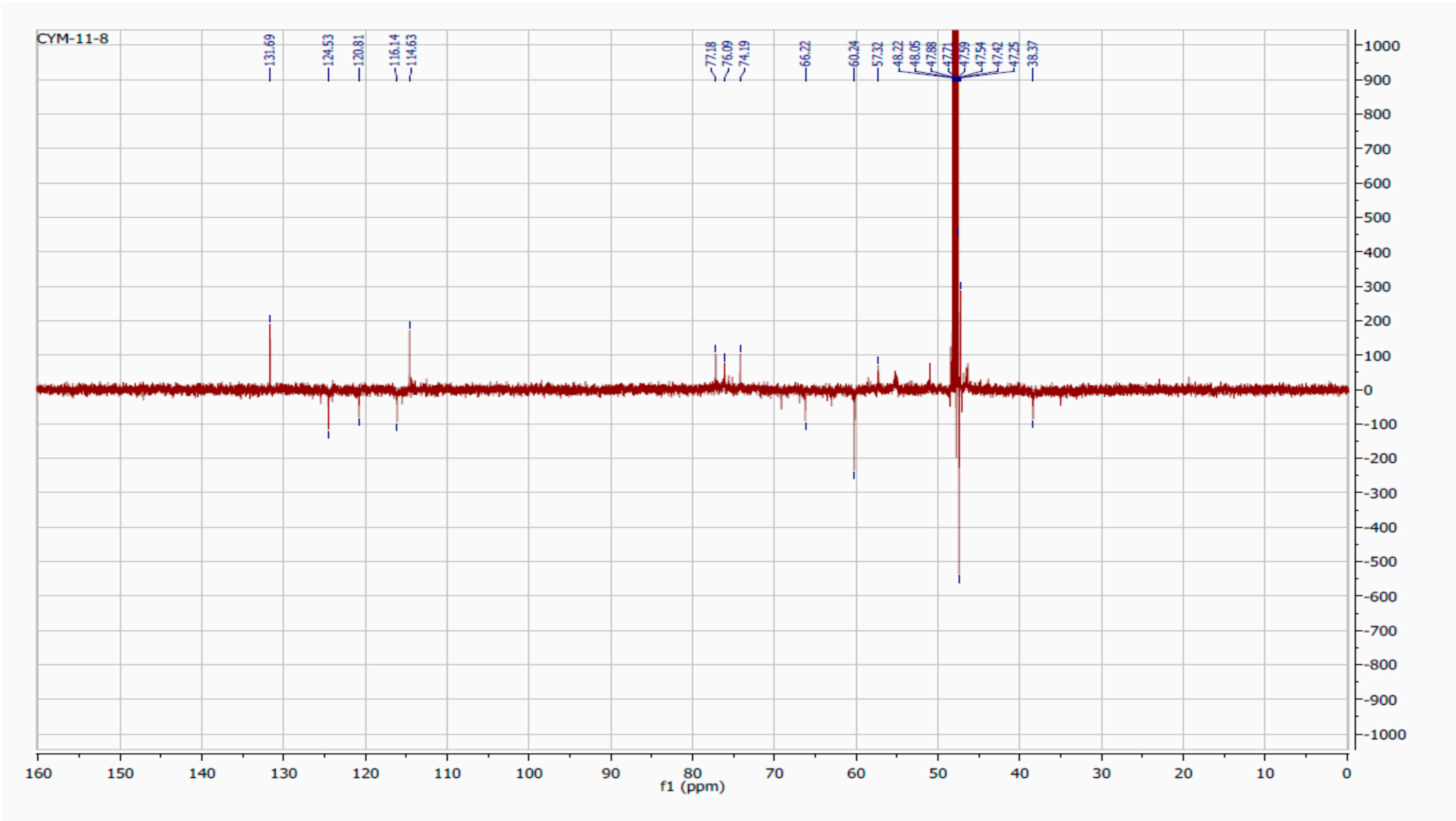
CYM-11-8
131.69
124.53
120.81
116.14
114.63
77.18
76.09
74.19
66.22
60.24
57.32
48.22
48.05
47.88
47.71
47.59
47.54
47.42
47.25
38.37
f1 (ppm)

S11:DEPT of **2**

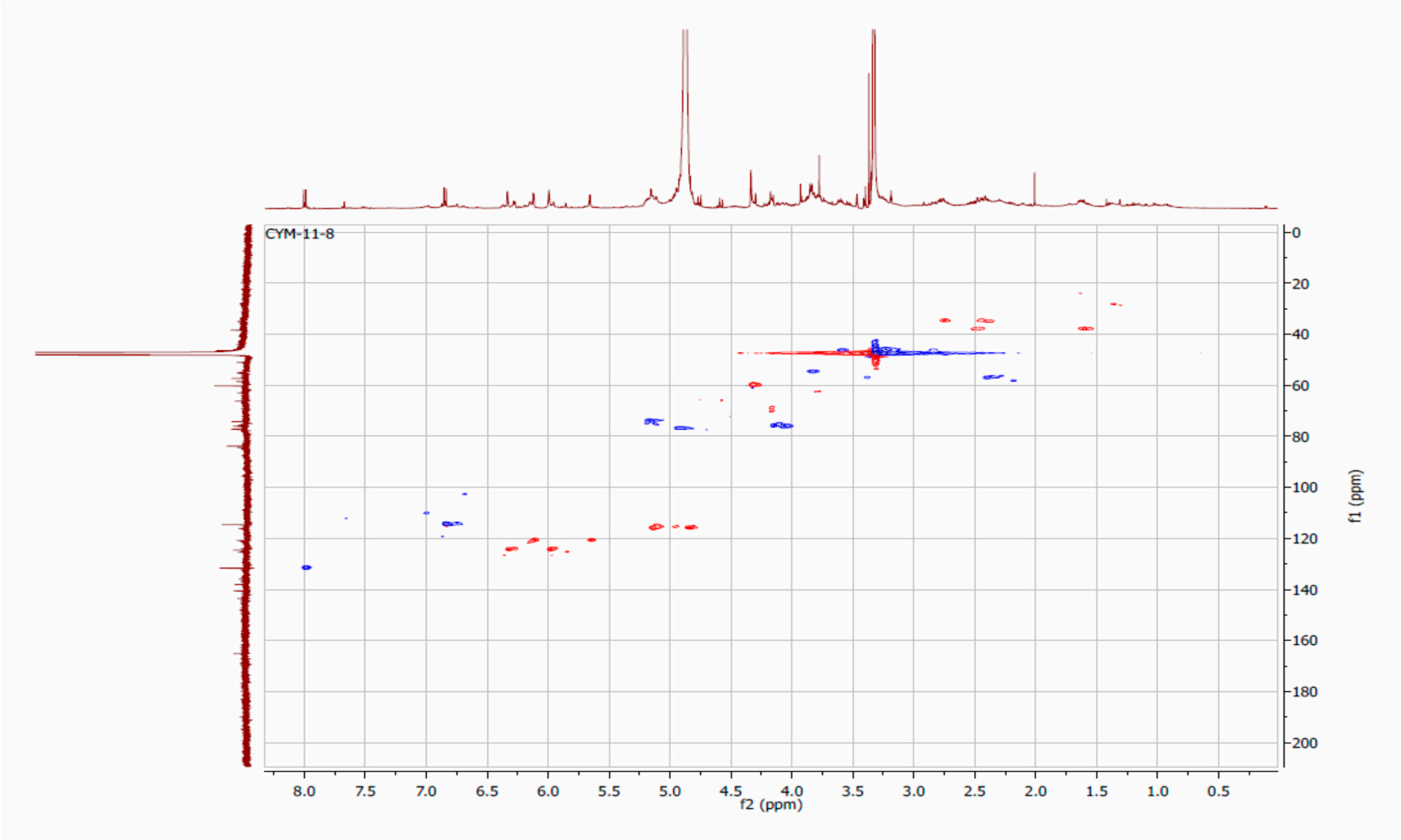

S12:HSQC of **2**

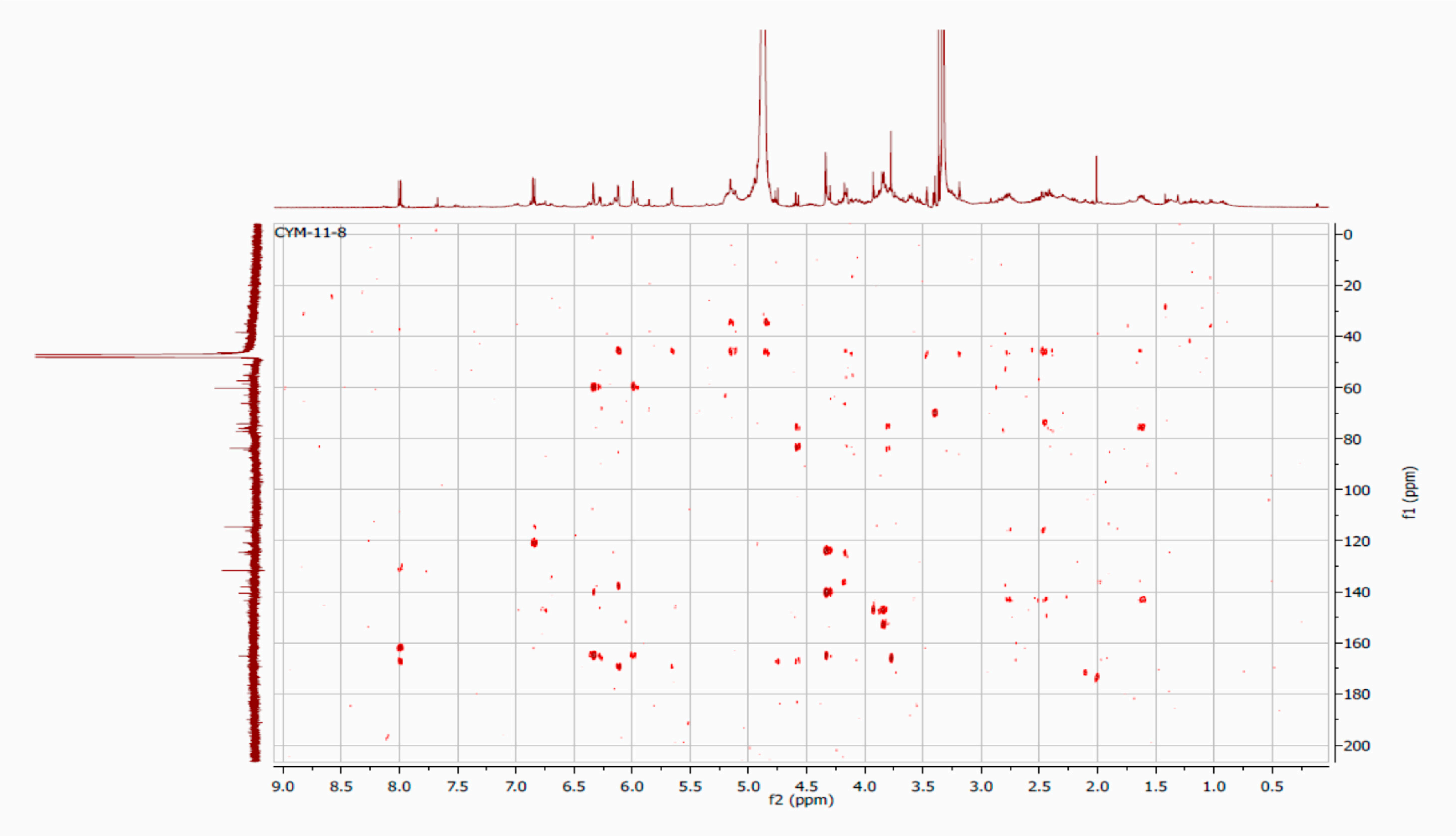
CYM-11-8
9.0
8.5
8.0
7.5
7.0
6.5
6.0
5.5
5.0
4.5
4.0
3.5
3.0
2.5
2.0
1.5
1.0
0.5
f2 (ppm)
0
20
40
60
80
100
120
140
160
180
200
f1 (ppm)

S13:HMBC of **2**

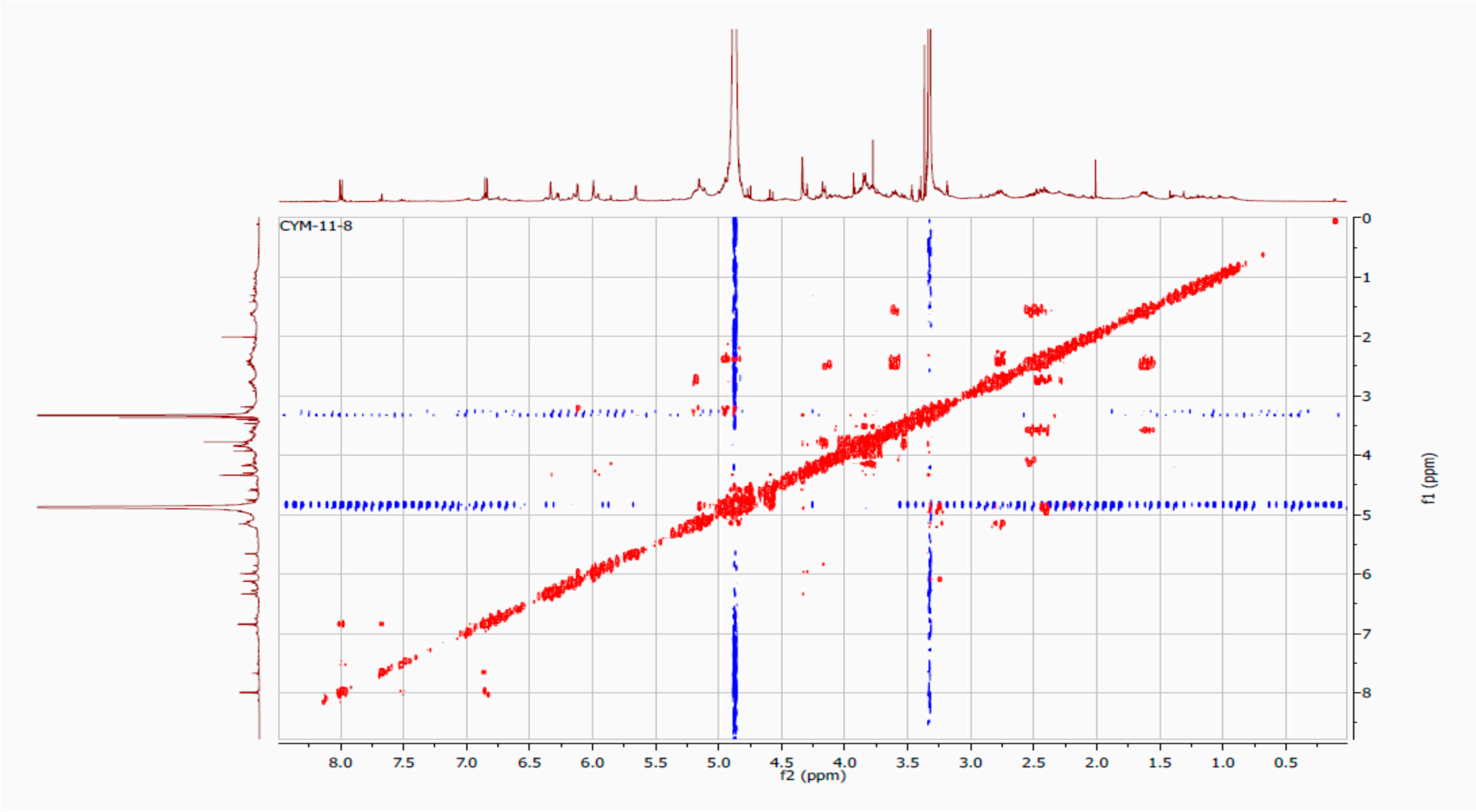

S14:^{1}H ^{1}H COSY of **2**

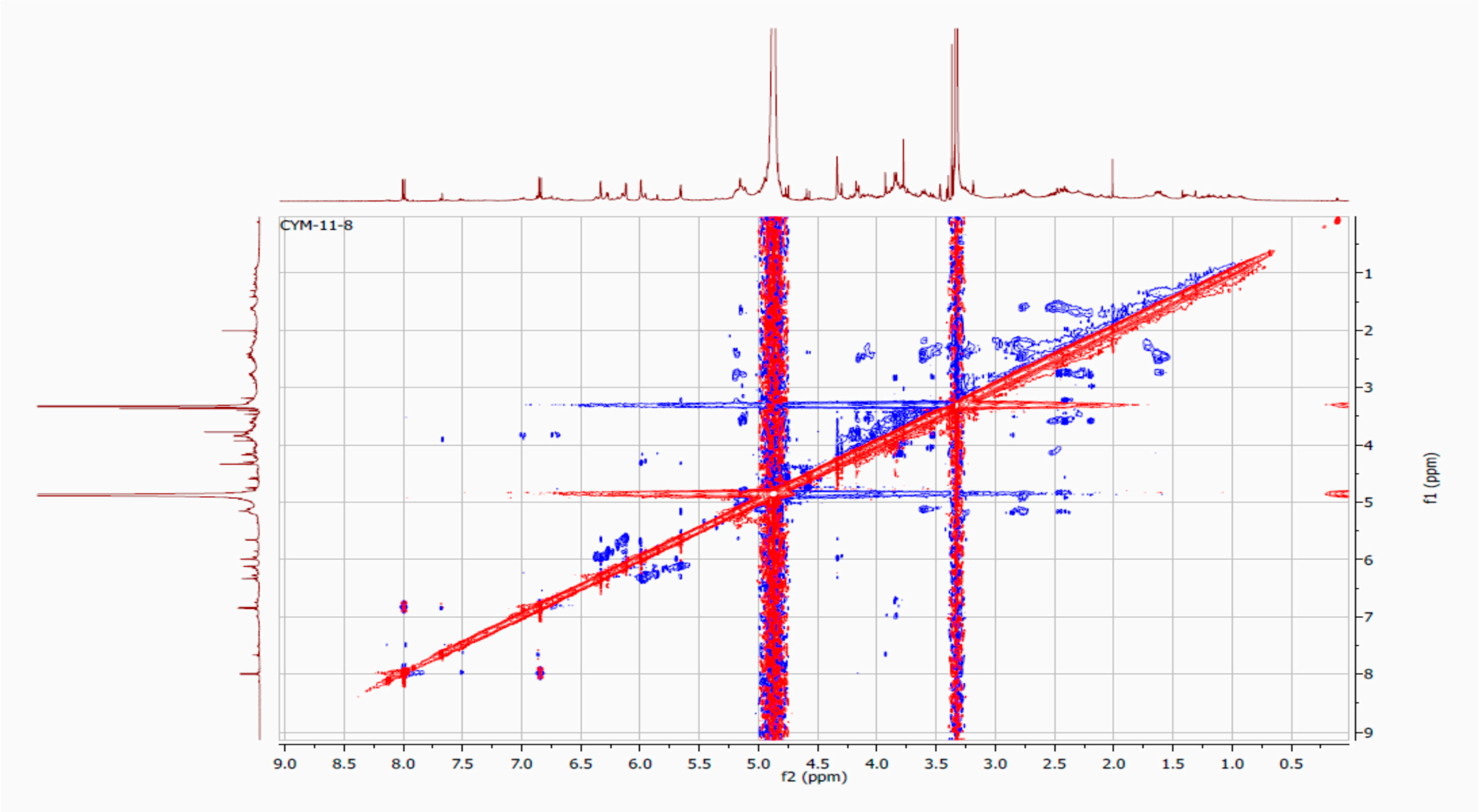

S15:NOESY of **2**

Sample : CyM-11-8
Note : MStation
Inlet : Direct   Ion Mode : CI+
RT : 2.26 min   Scan# : 60
Elements : C 150/0, H 250/0, O 50/0
Mass Tolerance   : 5mmu
Unsaturation (U.S.) : 0.0 – 15.0

	Observed m/z	Int%	Err[ppm / mmu]	U.S.	Composition
1	472.1758	1.34	+5.2 / +2.5	12.0	C25 H28 O9
2			–7.2 / –3.4	3.0	C18 H32 O14

S 16: HRCI/mas of **2**

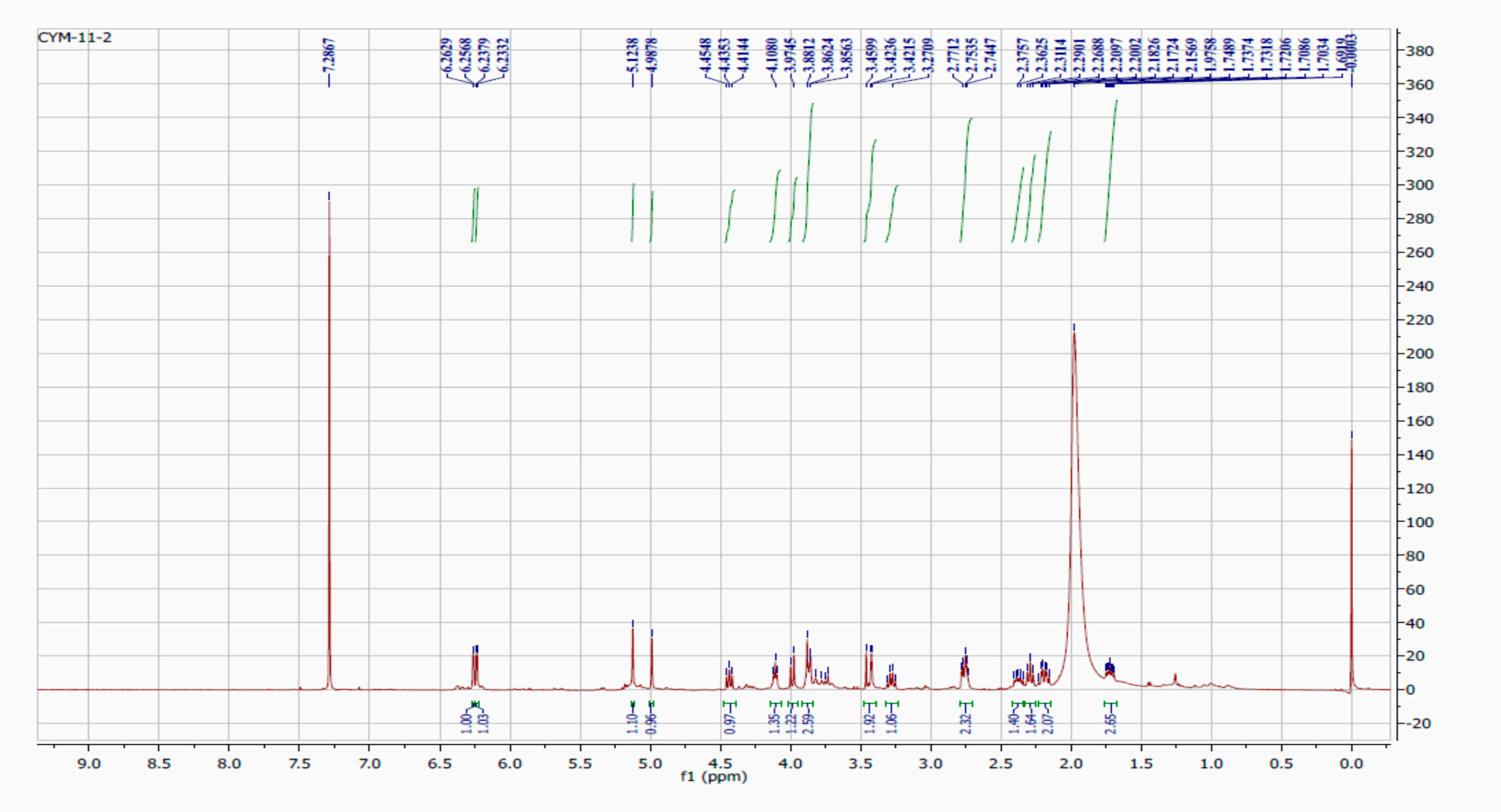

S17: ^1H NMR ($CDCl_3$, 500 MHz) of **3**

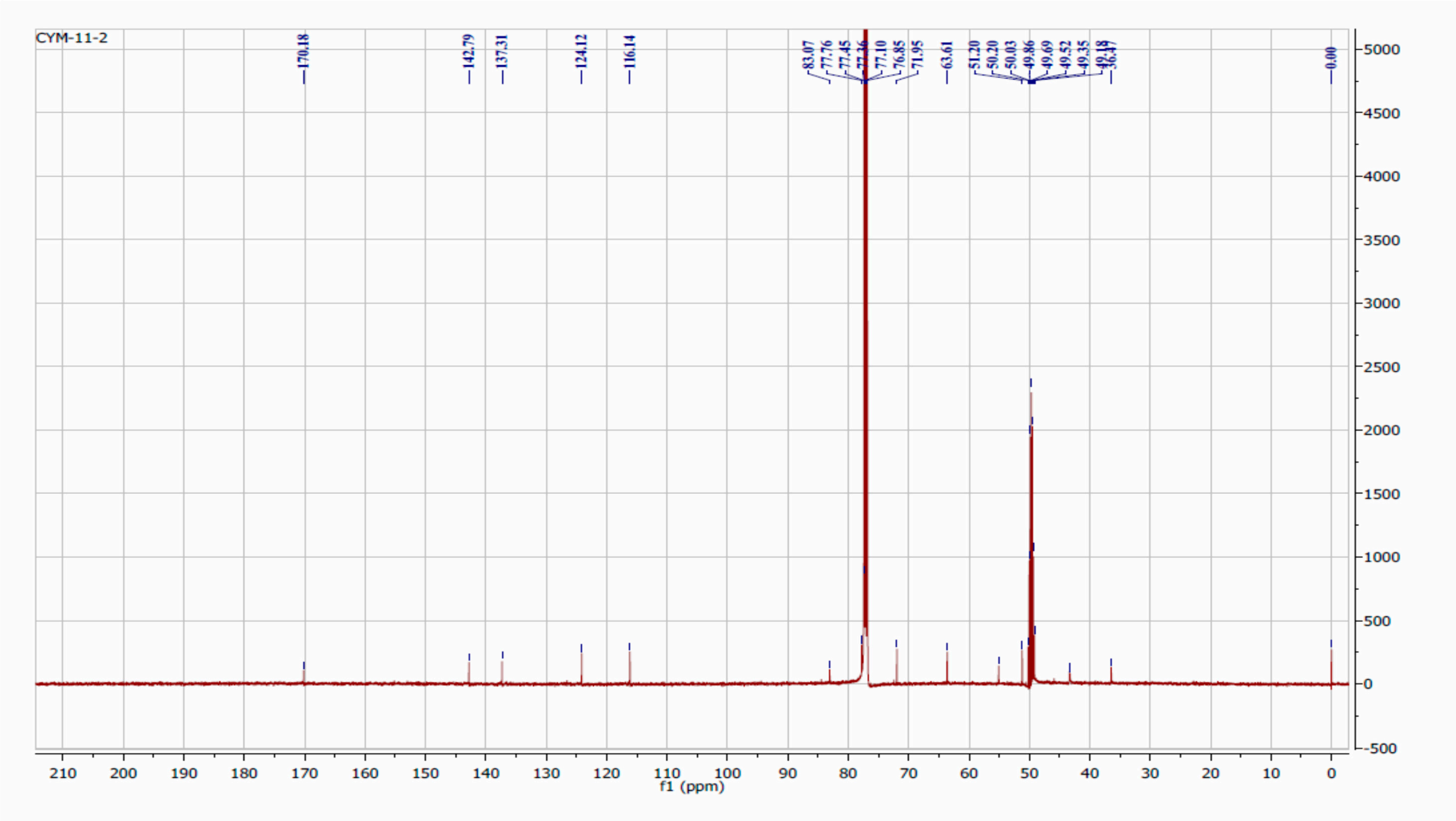

S18:^{13}C NMR (C_5D_5N, 125 MHz) of **3**

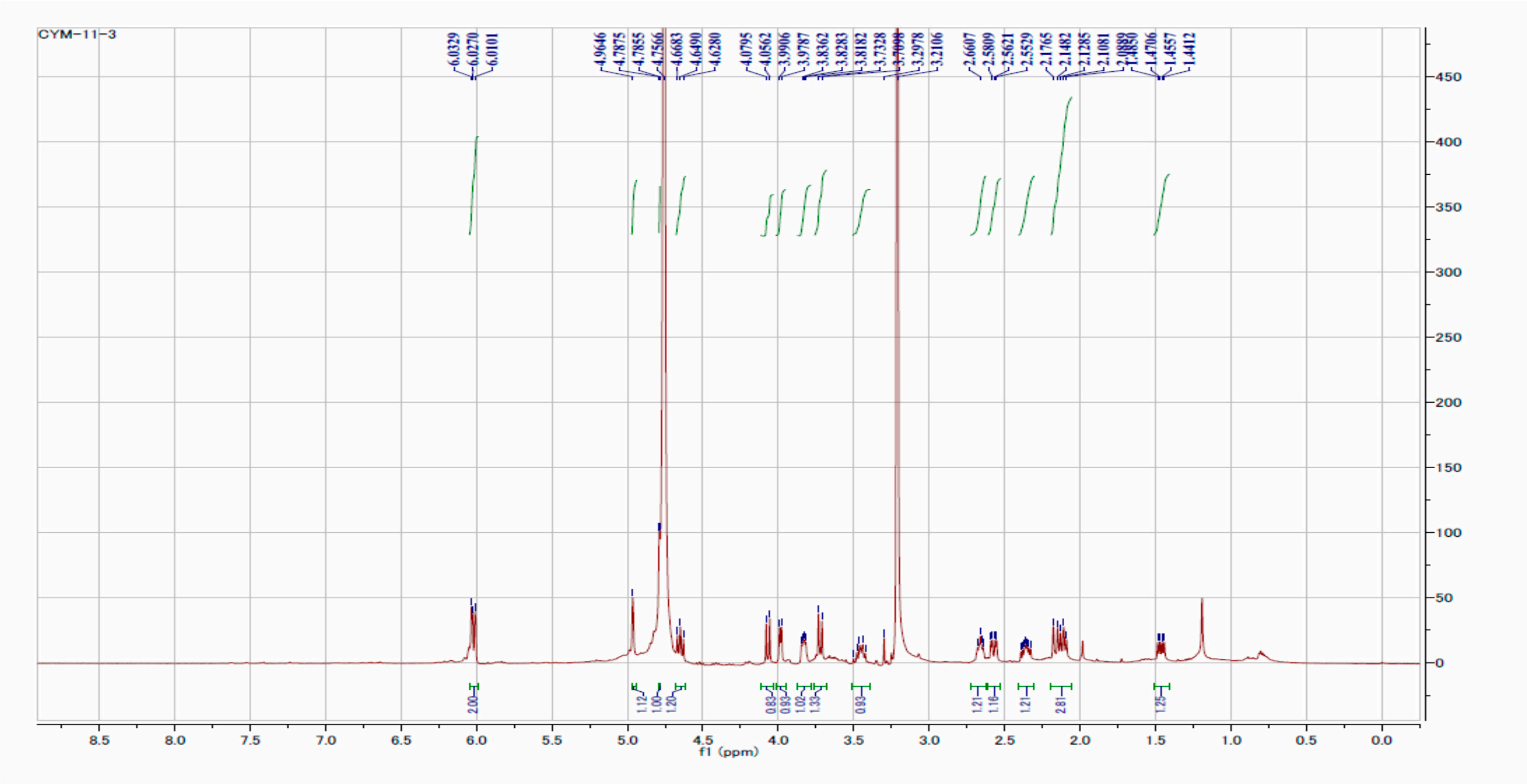

S19:^{1}H NMR ($CDCl_3$, 500 MHz) of **4**

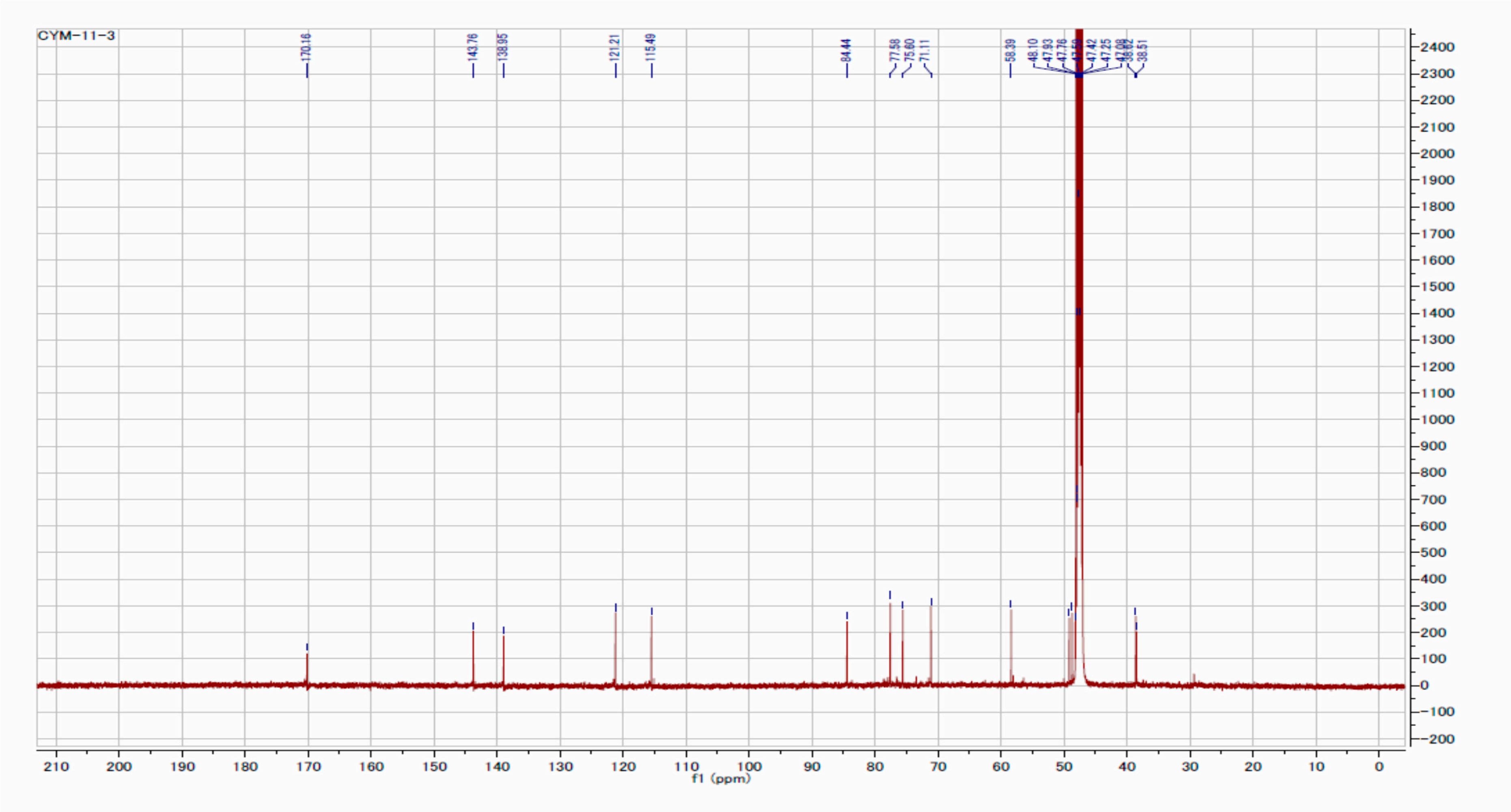

S20:^{13}C NMR (C_5D_5N, 125 MHz) of **4**

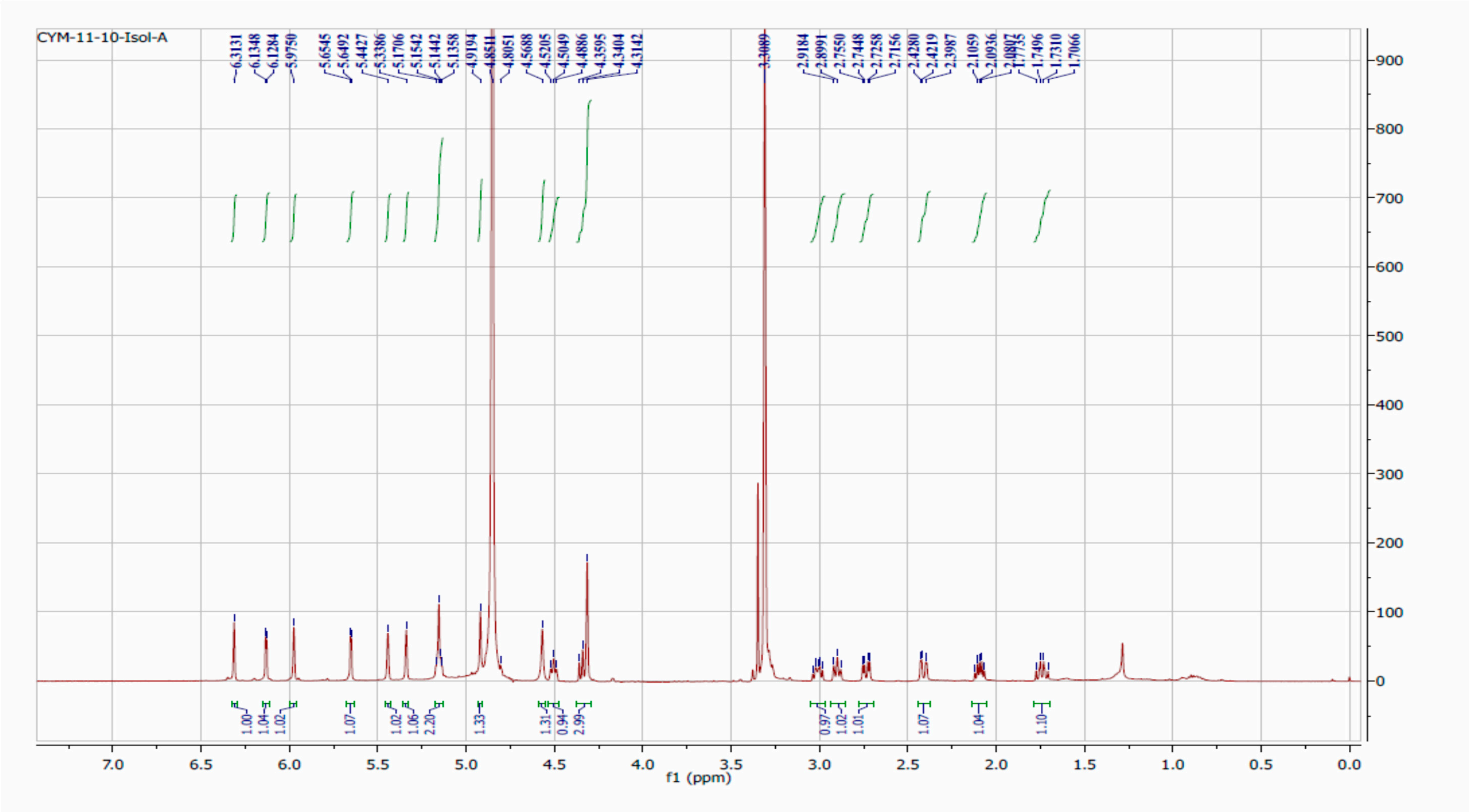

S21: ^{1}H NMR ($CDCl_3$, 500 MHz) of **5**

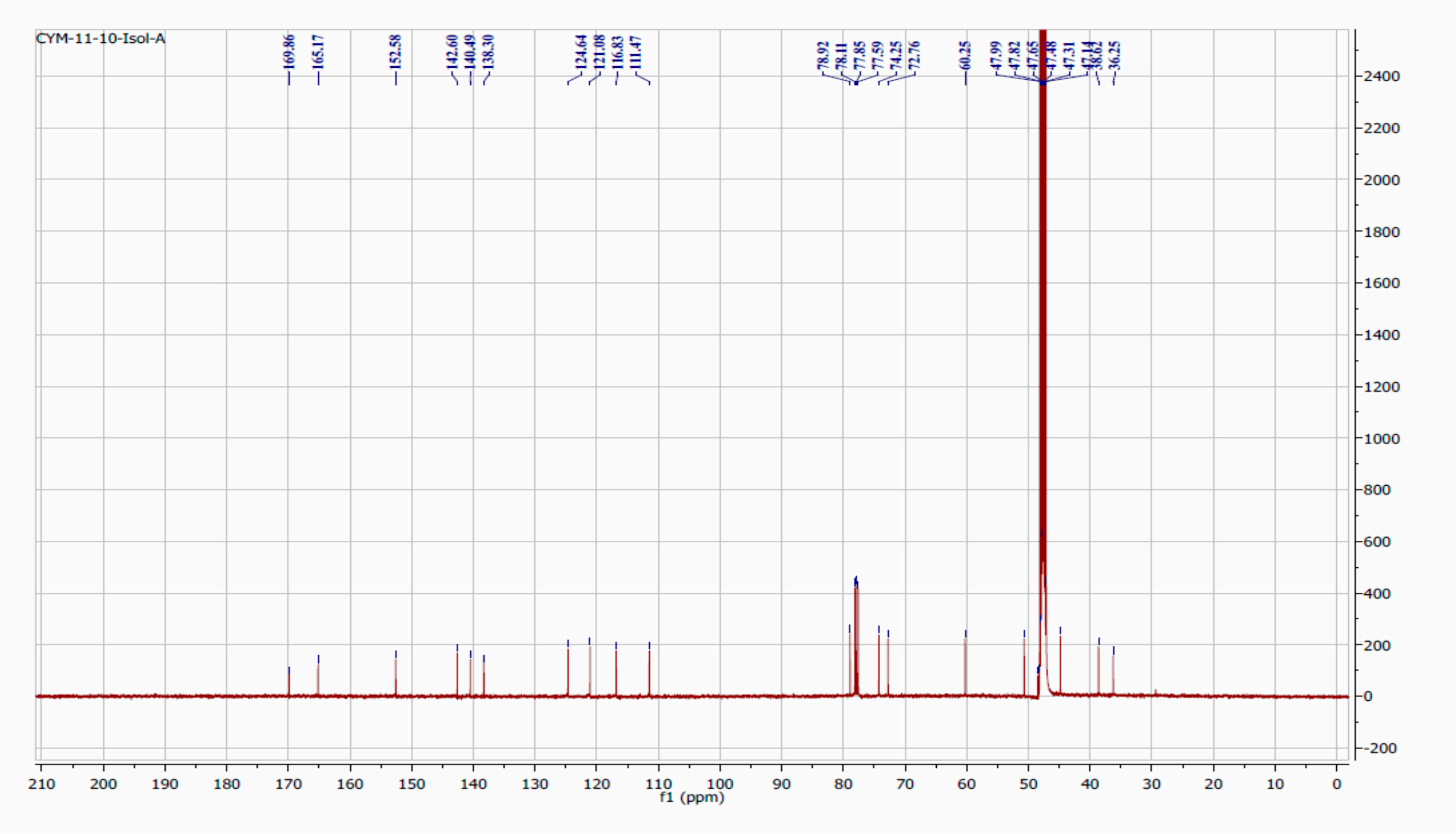

S22: ^{13}C NMR (C_5D_5N, 125 MHz) of **5**

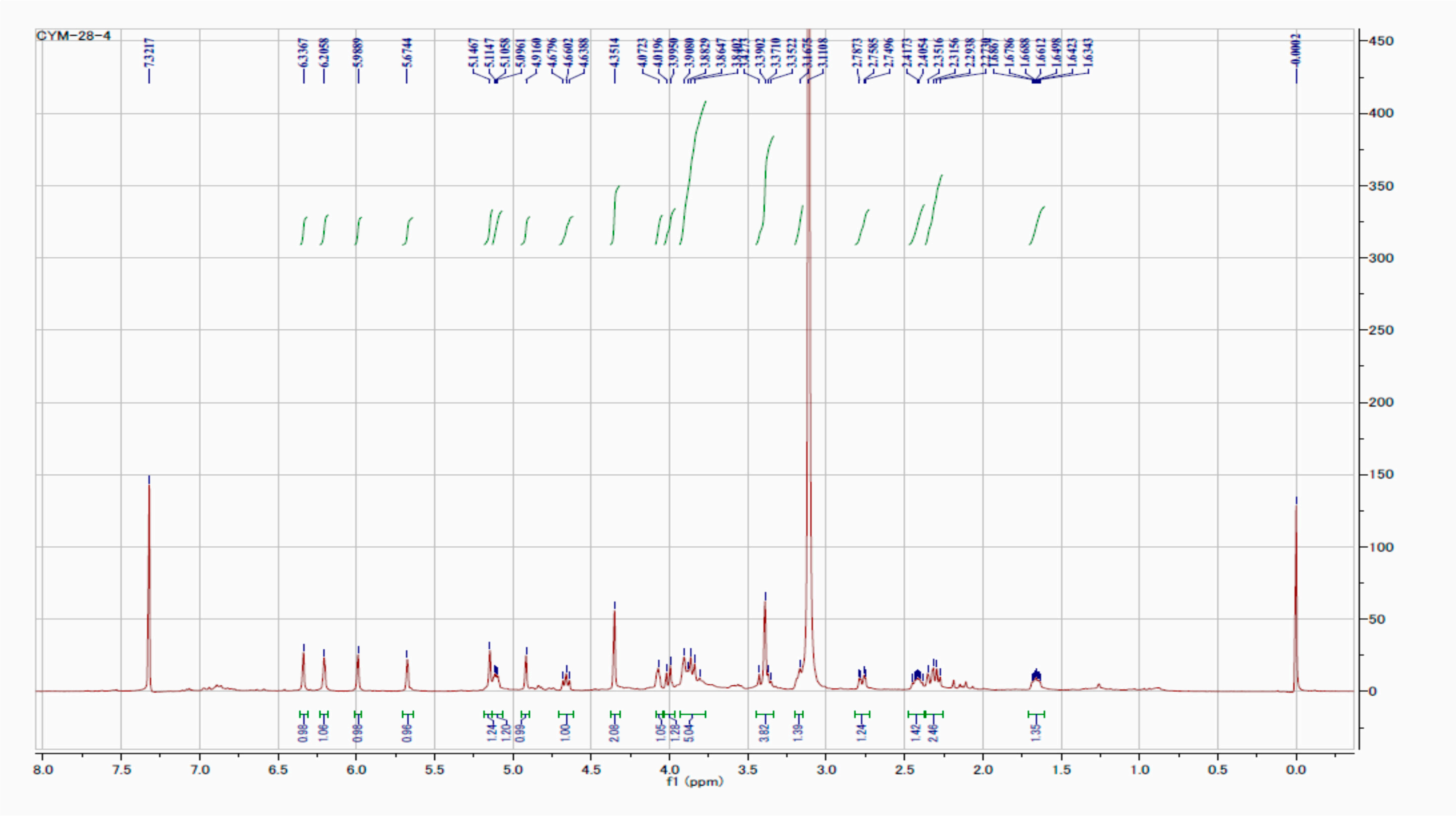

S23: ^{1}H NMR ($CDCl_3$, 500 MHz) of **6**

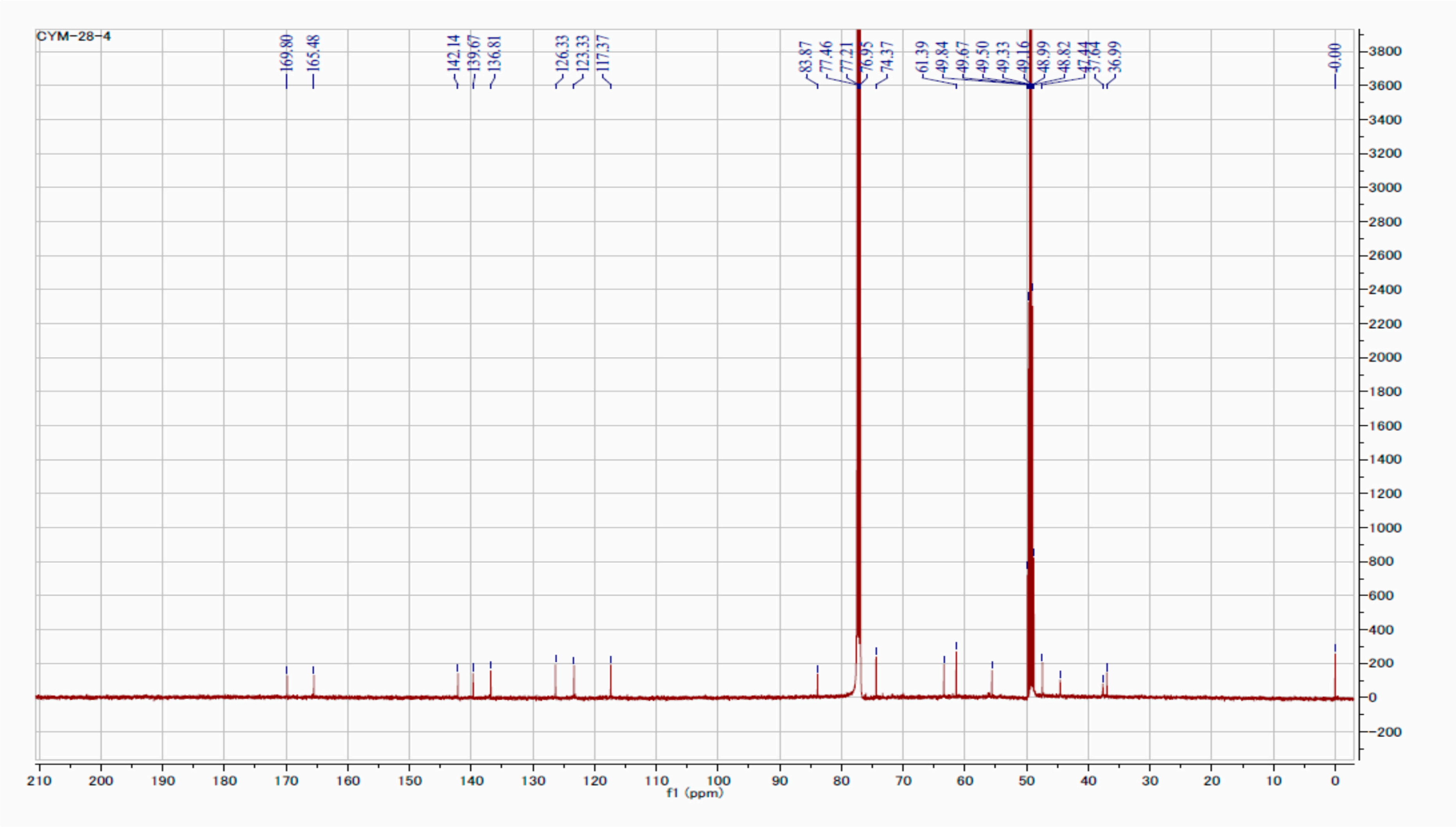

S24: ^{13}C NMR (C_5D_5N, 125 MHz) of **6**

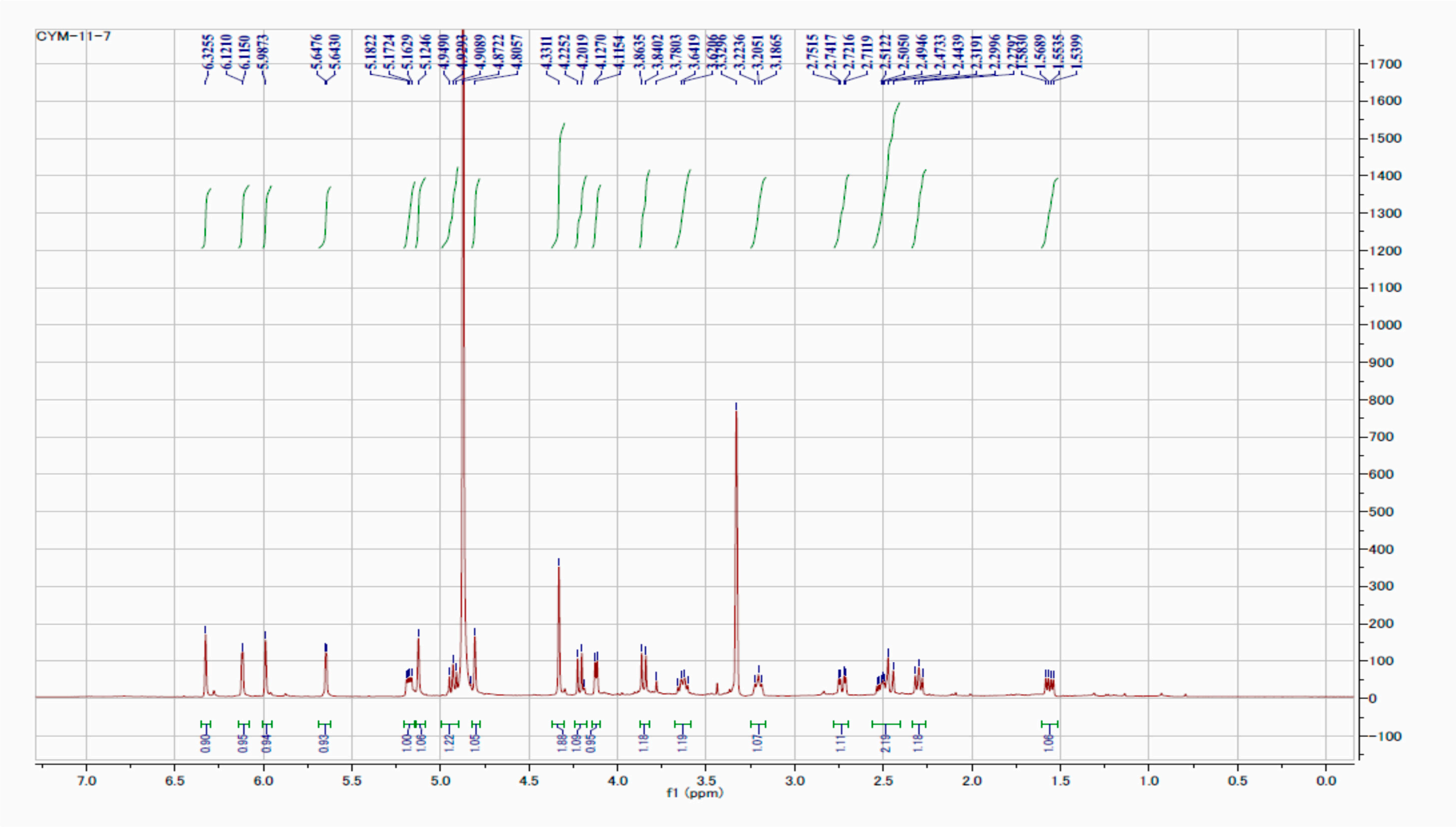

S25:^{1}H NMR ($CDCl_3$, 500 MHz) of **7**

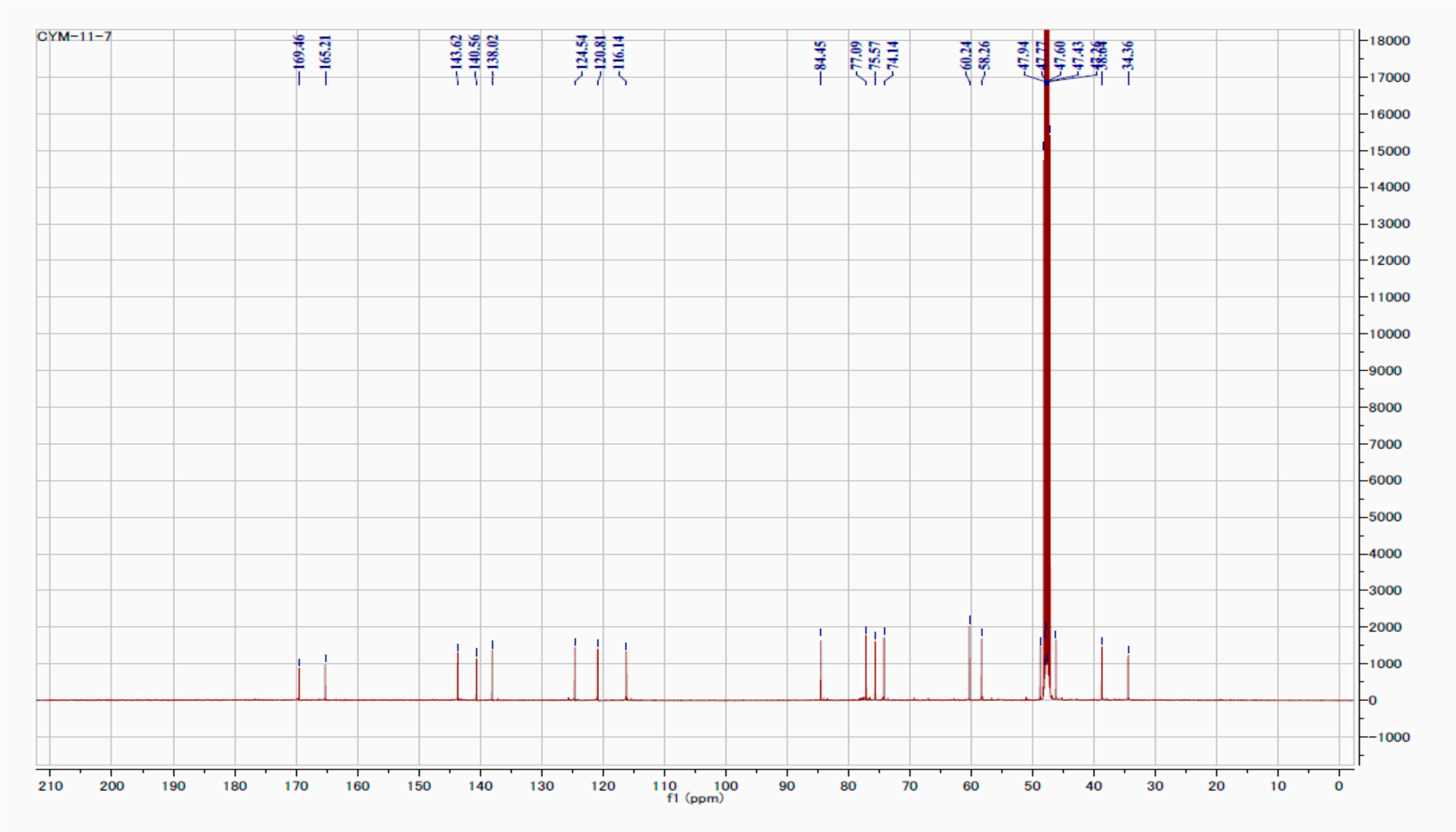

S26: ^{13}C NMR (C_5D_5N, 125 MHz) of **7**

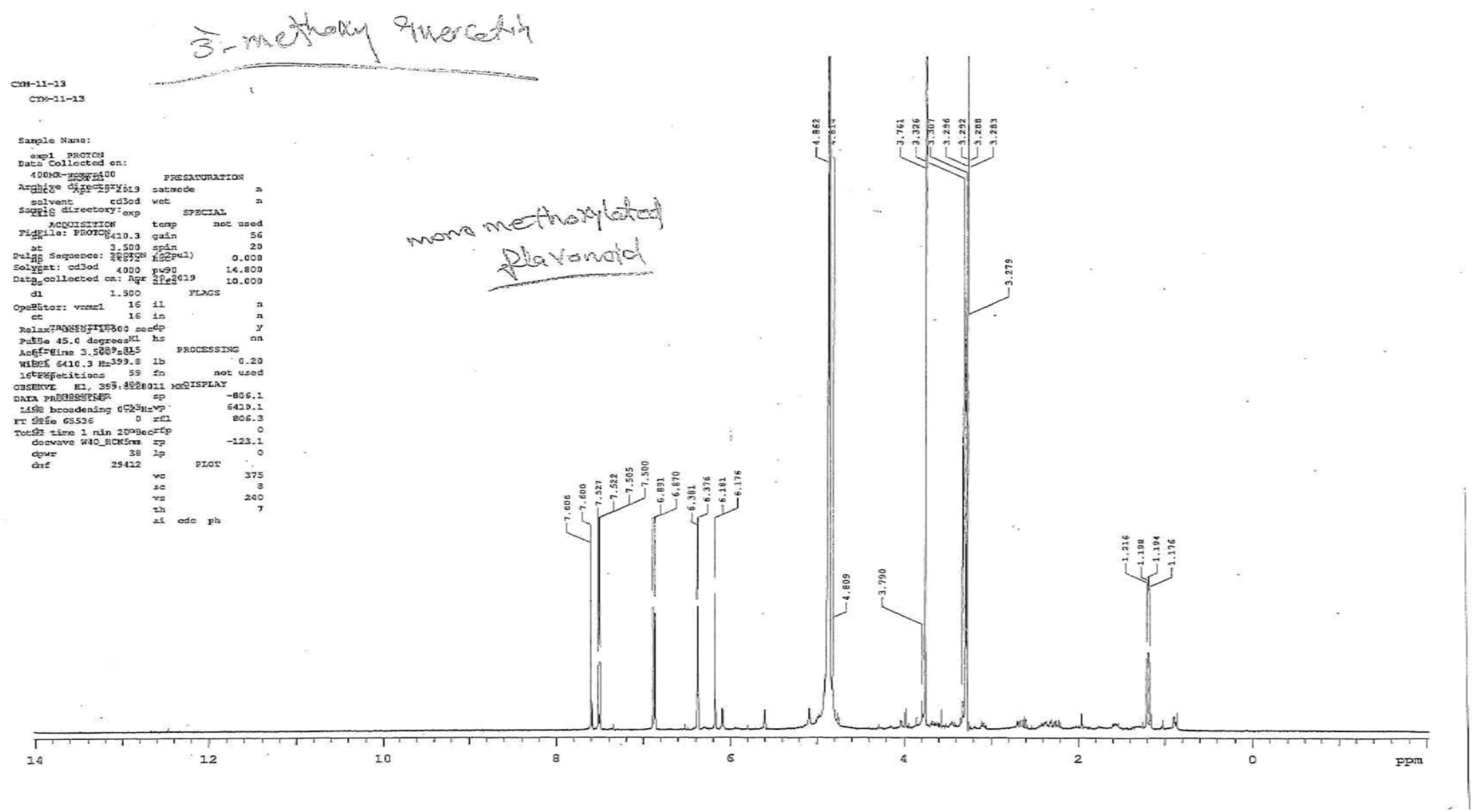

S27:^{1}H NMR ($CDCl_3$, 500 MHz) of **8**

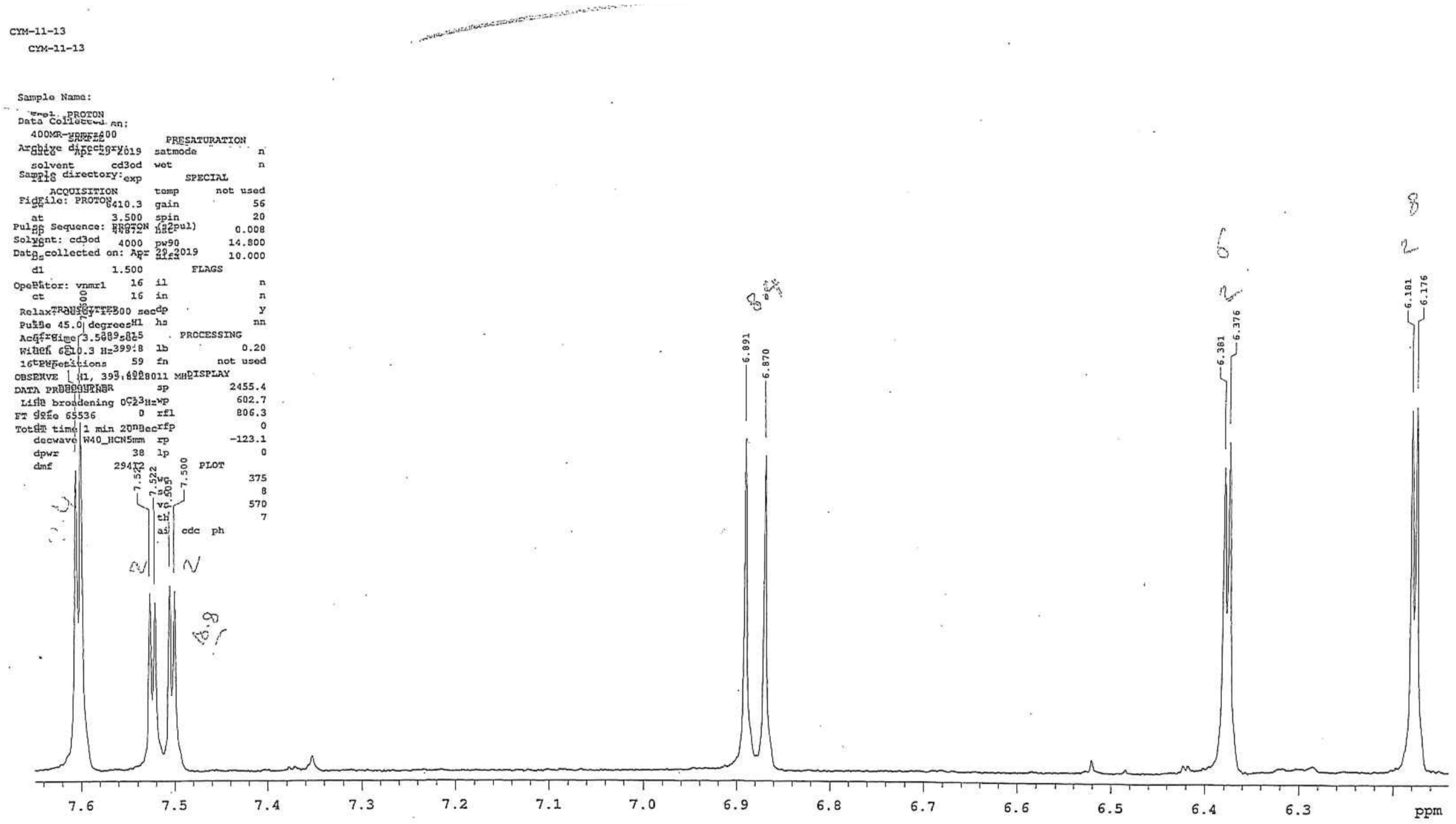

S27: ^{1}H NMR (C_5D_5N, 125 MHz) of **8**

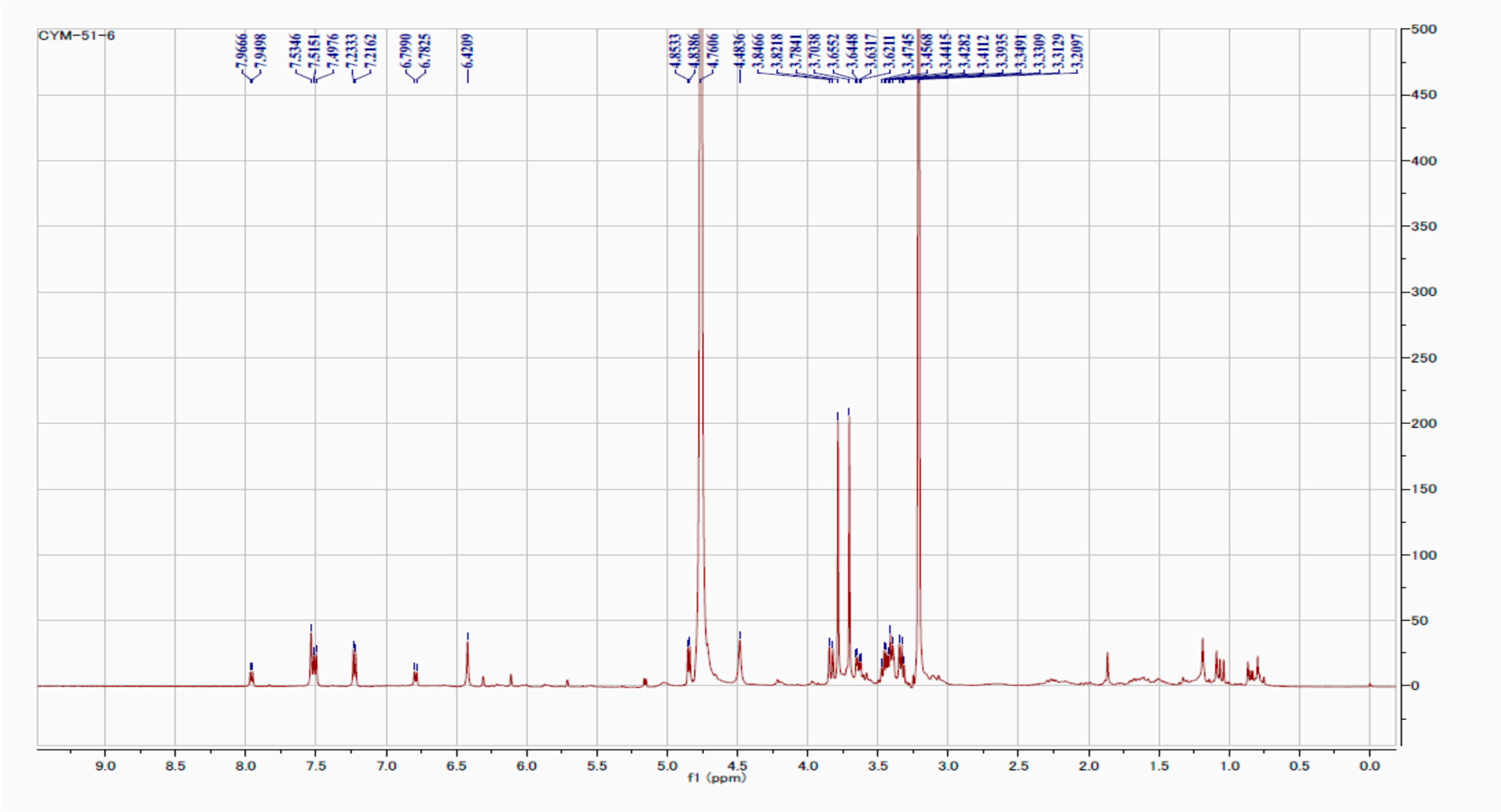

S28: ^{1}H NMR ($CDCl_3$, 500 MHz) of **9**
